# Expansion of EasyClone-MarkerFree toolkit for *Saccharomyces cerevisiae* genome with new integration sites

**DOI:** 10.1093/femsyr/foab027

**Published:** 2021-04-24

**Authors:** Mahsa Babaei, Luisa Sartori, Alexey Karpukhin, Dmitrii Abashkin, Elena Matrosova, Irina Borodina

**Affiliations:** The Novo Nordisk Foundation Center for Biosustainability, Technical University of Denmark, Kemitorvet Building 220, DK-2800 Kgs. Lyngby, Denmark; The Novo Nordisk Foundation Center for Biosustainability, Technical University of Denmark, Kemitorvet Building 220, DK-2800 Kgs. Lyngby, Denmark; Ajinomoto-Genetika Research Institute, Moscow, Russian Federation; Ajinomoto-Genetika Research Institute, Moscow, Russian Federation; Ajinomoto-Genetika Research Institute, Moscow, Russian Federation; The Novo Nordisk Foundation Center for Biosustainability, Technical University of Denmark, Kemitorvet Building 220, DK-2800 Kgs. Lyngby, Denmark

**Keywords:** chromosomal integration, CRISPR-Cas9, genome editing, metabolic engineering

## Abstract

Biotechnological production requires genetically stable recombinant strains. To ensure genomic stability, recombinant DNA is commonly integrated into the genome of the host strain. Multiple genetic tools have been developed for genomic integration into baker's yeast *Saccharomyces cerevisiae*. Previously, we had developed a vector toolkit EasyClone-MarkerFree for stable integration into eleven sites on chromosomes X, XI, and XII of *S. cerevisiae*. The markerless integration was enabled by CRISPR-Cas9 system. In this study, we have expanded the kit with eight additional intergenic integration sites located on different chromosomes. The integration efficiency into the new sites was above 80%. The expression level of green fluorescence protein (*gfp*) for all eight sites was similar or above XI-2 site from the original EasyClone-MarkerFree toolkit. The cellular growth was not affected by the integration into any of the new eight locations. The eight-vector expansion kit is available from AddGene.

## Teaser

The authors introduce a new set of validated chromosomal loci for the stable integration of heterologous DNA.

## INTRODUCTION

Baker's yeast *Saccharomyces cerevisiae* is a eukaryotic model organism (Nielsen 2019; Matthews and Vosshall 2020). It is also an industrial workhorse for producing various chemicals ranging from low-value high-volume compounds as ethanol (Caspeta *et al*. 2014) to high-value low-volume products as insulin (Liu *et al*. 2012). The development of industrial strains usually requires multiple iterative rounds of genome editing and metabolic rewiring of the host cell (Nielsen and Keasling 2016). Therefore, it is essential to have genetic toolkits that enables deletions, mutations, and integrations. The critical requirements for integration toolkit are: (i) stable chromosomal integration of DNA constructs, (ii) a high level of heterologous gene expression and (iii) no negative effects on the cellular fitness and growth (Gu *et al*. 2015).

To meet these requirements, our group has previously developed the vector toolkit EasyClone MarkerFree (Jessop-Fabre *et al*. 2016) for chromosomal integration of multiple genes into laboratory and industrial strains of *S. cerevisiae* (Jensen *et al*. 2014; Stovicek *et al*. 2015) using standardized cloning plasmids. This method provides stable integration and high expression of fragments into 11 individual sites located in intergenic regions in chromosomes X, XI and XII. When the same gene expression cassette was integrated into different sites, the expression levels were similar. Furthermore, the sites are separated by essential genes, which prevents the loss of integrated fragments by recombination and ensures strain stability (Mikkelsen *et al*. 2012). Selection markers are only present on two episomal plasmids, one for Cas9 expression, and another for helper gRNA expression. As no selection markers are integrated in the genome, multiple genome edits can be done consecutively without excising selection markers from the genome. For industrial-scale production, it is also essential that the production strain does not contain antibiotic resistance markers (Ramakrishnan *et al*. 2020).

In this study, we aimed to expand this toolkit with additional validated integration sites located on different chromosomes. The expansion is comprised of eight gRNA vectors targeting specific intergenic sites and of eight corresponding integrative vectors that can readily integrate into these sites by homologues recombination. The kit is available from AddGene (Deposit 78698).

## MATERIAL AND METHODS

gRNA plasmids targeting single and multiple integration locus were constructed as previously described (Jessop-Fabre *et al*. 2016). The chromosomal coordinates and nucleotide sequences of guide RNAs are listed in Table S1 (Supporting Information), with multiple gRNA vectors in Table S2 (Supporting Information). To construct the backbone plasmids, ca. 400–600 bp-long upstream and downstream regions of the target integration site (excluding PAM sequence) were PCR-amplified (Tables S5 and S6, Supporting Information). The UP and DOWN fragments were then USER-cloned with terminators T*adh1*-T*cyc1* (BB4368) and vector backbone for propagation in *E. coli* (BB4367). The vectors, listed in Table S3 (Supporting Information), were verified by Sanger sequencing (Eurofins, Germany). To prepare the *gfp* integrative vectors, codon-optimized *gfp* protein from *Aequorea victoria* (GenBank: AMY56666.1) was cloned to backbone plasmids according to the workflow of EasyClone-MarkerFree method (Jessop-Fabre *et al*. 2016) to vectors listed in Table S4 (Supporting Information).

*Saccharomyces cerevisiae* strain CEN.PK113-7D obtained from Peter Kötter (Entian and Kötter 2007) was used as the basic strain for designing and constructing the toolkit. To express Cas9 protein, CEN/ARS replicon-containing vector pCfB2312 (P*tef1*-Cas9-T*cyc1*_kanMX), Addgene #78231, was transformed to CEN.PK113-7D strain, and selected on media containing 200 mg/L G418. The obtained strain (ST7574) was used as the parent for all the following constructed strains. All the yeast transformation was accomplished *via* lithium acetate method, using 1–2 µg of NotI-digested and gel-purified integrative plasmid together with 500 ng of gRNA vector using the previously described protocol (Jessop-Fabre *et al*. 2016). For verification of correct integration of vectors into the targeted site, a colony PCR using primers listed in Table S7 (Supporting Information) with RedTaq® DNA polymerase (VWR, Belgium) was applied. To evaluate the cell growth of strains with integrated *gfp* cassette into Expanded-Easyclone sites, 8–10 single colonies were cultivated in 500 µL mineral medium (Babaei *et al*. 2020) supplemented with 20 g/L glucose in 96-deep well plate. The next day, the cells were diluted 1:100 into 96-half deep-well plate containing 300 µL of mineral media and cultivated with shaking at 250 rpm and 30°C in Growth Profiler 960 (EnzyScreen, The Netherlands). The cultivation was continued for 40 h to measure the growth profile of the engineered strains. To study the stability of constructed cells, the culture was passaged every 24 hours to fresh medium with a dilution of 1:50. The *gfp* fluorescence and OD_600_ values were measured by BioTek Synergy MX microplate reader, with excitation at 485 nm, and emission at 530 nm for fluorescence.

## RESULTS AND DISCUSSION

**Figure 1.**
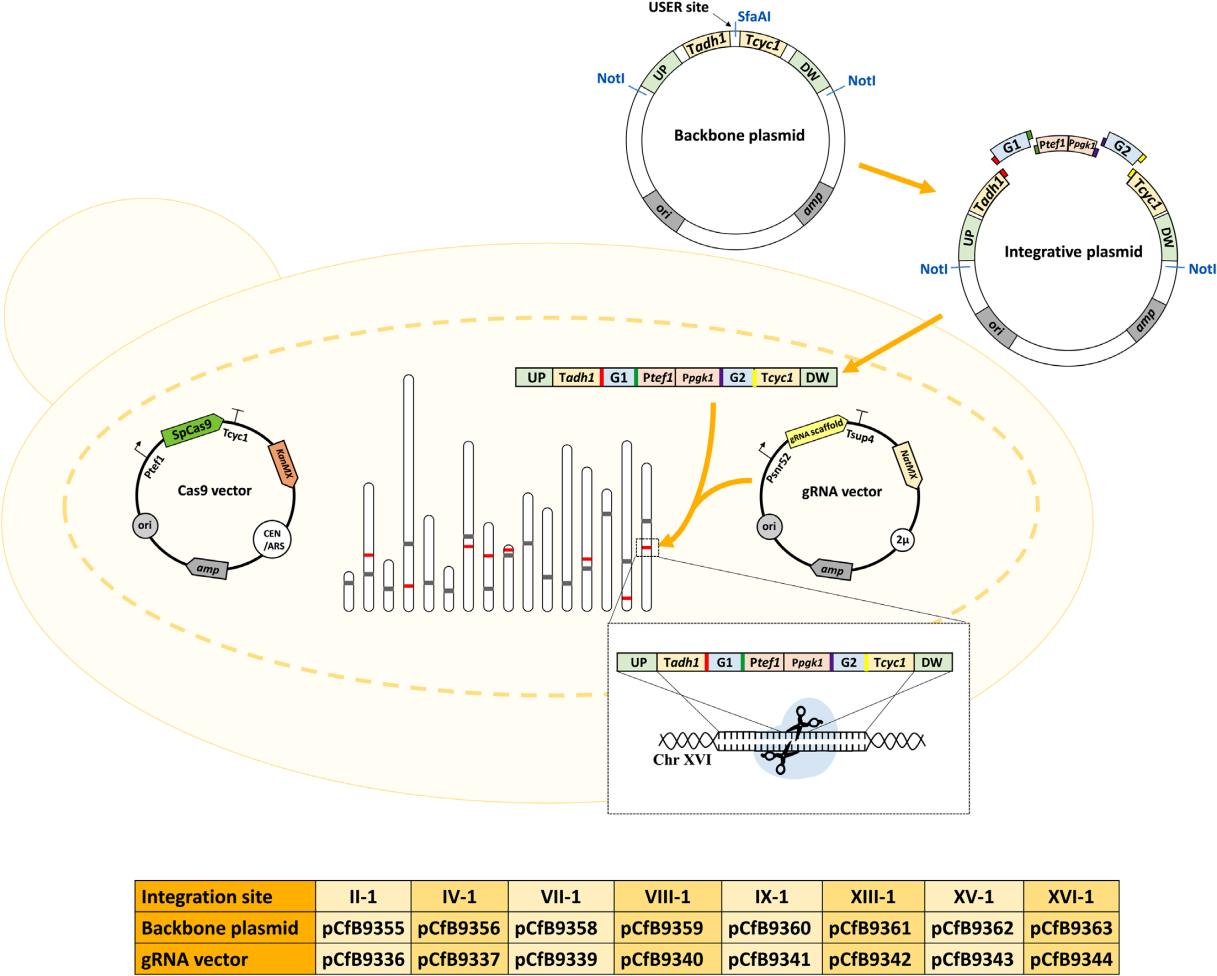
The Expanded EasyClone-MarkerFree toolkit.

When selecting new integration sites in the genome of *S. cerevisiae*, the intergenic (non-coding) regions meeting the following criteria were considered: (i) longer than 1500 nucleotides in length, (ii) without autonomous replicon sequence (ARS) and Ty elements and (iii) located far from the centomere and the telomeres (Mikkelsen *et al*. 2012). Eight potential integration sites meeting these criteria were selected and named after the chromosome number (Table S1, Supporting Information), as II-1, IV-1, etc. The gRNA sequences were then designed to target these integration sites in *S. cerevisiae* CEN.PK113-7D strain. This set of integrative expression vectors allows marker-free integration of one or two gene expression cassettes in each standardized cloning vector (Fig. 1). The workflow in the expanded toolkit is designed to be similar and compatible with the original EasyClone-MarkerFree toolkit, described by Jessop-Fabre et al (Jessop-Fabre *et al*. 2016). The co-transformation of gRNA plasmids together with NotI-linearized integrative vectors into the yeast cell expressing Cas9 protein results in integration of the cassette into the specific chromosomal site. To screen for positive transformants, a quick and reliable yeast colony PCR using the primers listed in Table S7 (Supporting Information) can be used. The yeast cells with correct integration would then be cured for gRNA vector by plating the cells in non-selective medium lacking nourseothricin. If at the same time, the selection for Cas9-plasmid with G418 resistance marker is maintained, then the resulting cell will be ready for the next round of integration. At the end, the strain is plated on the non-selective medium to remove both the gRNA and the Cas9 vector.

The verification of the integration efficiency and gene expression level for the new sites was accomplished by integration of *gfp* into each site. To do this, we cloned *gfp* expression cassette, with two different sets of promoters-terminators; P*tef1*-*gfp*-T*adh1*, and P*pgk1*-*gfp*-T*cyc1*, to obtain integrative plasmids (pCfB9365-pCfB9373 and pCfB10514-pCfB10521, respectively; Table S4, Supporting Information). At the same time, we also constructed strains with double and triple insertion of *gfp* integration cassettes into different sites in a single-step transformation. To do the later, we constructed multiple gRNA vectors using the same method as before (Jessop-Fabre *et al*. 2016) (Table S2, Supporting Information). The plasmids were then integrated into CEN.PK113-7D strain expressing Cas9 (ST7574).

**Figure 2.**
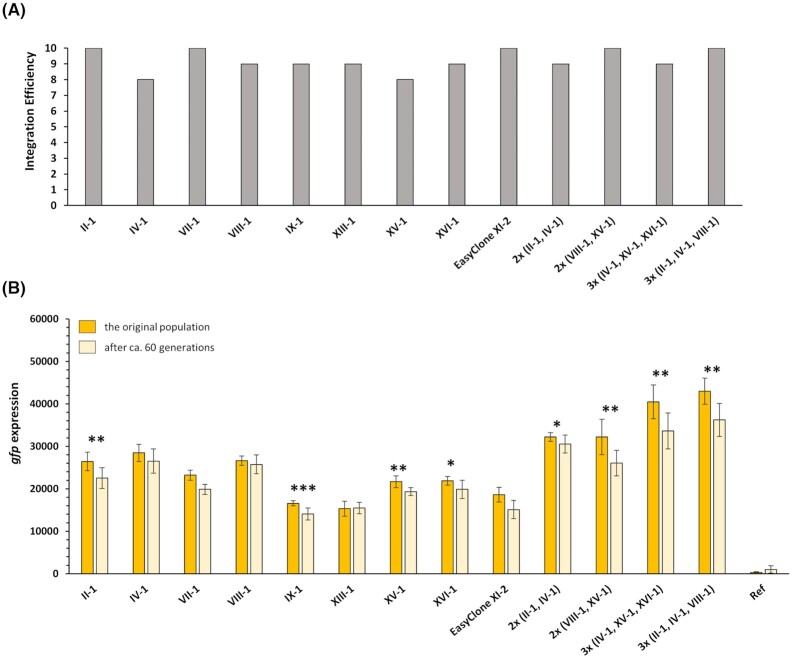
Characterization of EasyClone-expanded integrative vectors. **A**) Transformation efficiency shown as number of clones with correctly integrated DNA out of 10 randomly picked clones. **B**) Expression level of *Ptef1- gfp-*T*adh1* integrated into the new sites for original fluorescent population and these cells after cultivation for ca. 60 generations. Expression from EasyClone site XI-2 is shown for comparisson. The reported values for fluorescence are the mean for 8–10 replicates, with error bars showing standard deviation between the replicates. Statistical analysis was performed for *gfp* expression values of each strain for original and 60th generations by using Student's t test (one-tailed, two-sample unequal variances; *p <0.05, **p <0.01, ***p <0.001).

For all the transformations, we checked whether the plasmids were integrated into the correct site by randomly picking ten colonies and performing colony PCR. The transformation efficiency for all the explored sites with single and multiple integration of P*tef1*-*gfp*-T*adh1* cassette was at least 8 out of 10 colonies or above, as shown in Fig. 2A. These numbers are comparable with the original EasyClone-MarkerFree vectors (Jessop-Fabre *et al*. 2016). To check for the selected gRNA sequences' efficiency, we also included negative control transformations, carried out with solely 500 ng gRNA vector and lacking the repair DNA. For all the integration sites, only very few colonies were obtained for the negative controls.

We then cultivated at least eight clones with correctly integrated P*tef1*-*gfp*-T*adh1* cassette for each single and multiple vector and measured fluorescence. The integration sites selected for expanded toolkit are located in different chromosomes, which is different from the original EasyClone toolkit with sites located on the same chromosomes but spaced by essential genetic elements. Therefore, to check whether the new sites are stable, we measured the expression level of *gfp* for constructed cells from transformation plate (termed as ‘original population’) and compared it to the fluorescence level of cells after multiple passages (to reach 60th generation). The *gfp* expression levels as shown in Fig. 2B for all explored integration sites were comparable or higher than the control EasyClone site XI-2, with additive effect of double and triple integration of *gfp* cassette in cell fluorescence (Fig. 2B). Furthermore, when we compared the *gfp* expression level after several passages of fluorescent cells with the original population, we observed a slight reduction in four sites; 14% in site II-1 (*p* < 0.01), 15% in site IX-1 (*p* < 0.001), 10% in site XV-1 (*p* < 0.01), and 9% in site XVI-1 (*p* < 0.05). For the cells with multiple integrated *gfp* in any of these sites, a similar trend in reduction of fluorescence was observed after 60 generations. For other sites, including Easyclone XI-2, the reduction in *gfp* expression was not significant (*p* > 0.05). Though the reduction in *gfp* expression in sites II-1, IX-1, XV-1 and XVI-1 was not drastic, it might be useful to consider this observation when constructing strains for processes with prolonged cultivations.

**Figure 3.**
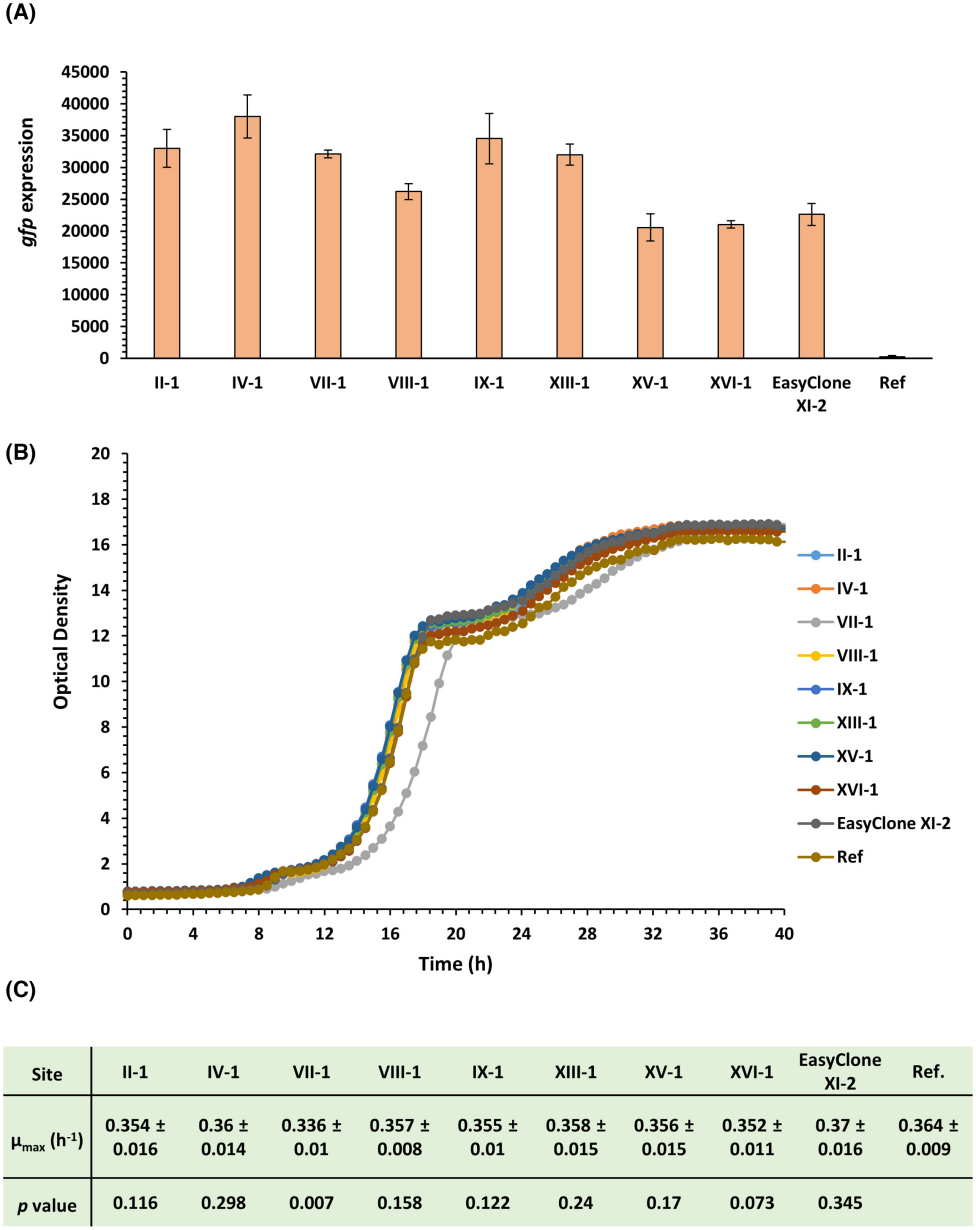
**A**) Expression level of P*pgk1- gfp-*T*cyc1* integrated into the new sites for cells after cultivation for ca. 60 generations. Expression from EasyClone site XI-2 is shown for comparisson. **B**) Growth profiles of strains with integrated *gfp* cassette, compared with non-transformed strain ST7574 as reference (Ref). **C**) Maximum specific growth rates (µ_max_, h^−1^) for the growth profiles of strains in panel B. Statistical analysis was performed for µ_max_ values of each strain and reference by using Student's t test (one-tailed, two-sample unequal variances).

To investigate the performance of the new integration sites using a different combination of regulatory elements, we constructed the cells with *gfp* integration cassette with a constitutive promoter P*pgk1* and terminator T*cyc1* (P*pgk1*-*gfp*-T*cyc1*). Similar to the previous results (Fig. 2B, for P*tef1*-*gfp*-T*adh1*), the *gfp* expression level for P*pgk1*-*gfp*-T*cyc1* integration to all the sites were similar or higher than EasyClone XI-2 (Fig. 3A). When comparing the expression levels of *gfp* between the sites for the two different promoter constructs (Fig. S1, Supporting Information), the sites II-1, IV-1, VII-1 and VIII-1 gave consistently higher expression level than EasyClone XI-2 site, while sites XV-1 and XVI-1 resulted in a similar expression level as EasyClone XI-2 site. However, *gfp* expression in sites IX-1 and XIII-1 was dependent on the promoter. As expression level of a protein depends on many factors in addition to the regulatory elements and integration site, it is necessary to optimize the expression empirically. Next, to ensure that the integration of foreign DNA elements into the explored genomic sites does not influence the cell growth, we also measured the later and compared it with the reference strain without any integrations (Fig. 3B). Except for the strain with *gfp* integrated into site VII-1, which had a 2-hour longer lag phase, all the other strains had growth characteristics similar to the reference strain (ST7574). In terms of the maximum specific growth rate (µ_max_) shown in Fig. 3C, this value for all the strains was similar to the reference strain (*p* > 0.05), except for strain of site VII-1 (*p* < 0.01). As the transformants used for growth analysis were not cured for gRNA vector (harboring antibiotic-resistant marker NatMX), the observed lower µ_max_ in growth for site VII-1 might be retrieved after few cultivations in non-selective media.

In conclusion, the expansion vectors offer the same integration efficiency, expression levels, and no impact on growth as the original vectors from the EasyClone-MarkerFree toolkit. We hope that they will be a useful addition to the genome editing tools available for the engineering of *S. cerevisiae*.

## AUTHORS’ CONTRIBUTION

IB and EM conceived the study. AK and DA designed the gRNA elements and performed preliminary tests. MB designed the toolkit plasmids and strains, and performed the growth profiler experiments. LS constructed vectors and strains and made *gfp* expression analysis. MB analyzed the data, and wrote the manuscript. All the authors contributed to writing and revising the manuscript. IB and EM secured the funding and supervised the project.

## Supplementary Material

foab027_Supplemental_File
