# Supplementary material for: Expansion of EasyClone-MarkerFree toolkit for Saccharomyces cerevisiae genome with new integration sites
Source: FEMS Yeast Res. 2021 Apr 24;21(4):foab027. doi: 10.1093/femsyr/foab027 (PMC8112480; doi:10.1093/femsyr/foab027)
Supplement: foab027_Supplemental_File [file foab027_supplemental_file.docx]

Supporting Information

Expanding EasyClone-MarkerFree toolkit, Evaluation of new integration sites in *Saccharomyces cerevisiae* genome

Mahsa Babaei^1^, Luisa Sartori^1^, Alexey Karpukhin^2^, Dmitrii Abashkin^2^, Elena Matrosova^2^, , Irina Borodina^1^*

**^1^** The Novo Nordisk Foundation Center for Biosustainability, Technical University of Denmark, Kemitorvet Building 220, DK-2800 Kgs. Lyngby, Denmark

^2^ Ajinomoto-Genetika Research Institute, Moscow, Russian Federation

*Corresponding Author

Supplementary Table S1: Details of gRNA plasmids...………………………………………………………………………3

Supplementary Table S2: List of multiple gRNA plasmids…………………………………………………………………3

Supplementary Table S3: List of backbone plasmids………………………………………………………………………..4

Supplementary Table S4: List of integrative plasmids for *gfp*...……………………………………………………..…5

Supplementary Table S5: List of BioBricks………………………………………….…………………………………………….6

Supplementary Table S6: List of Primers…………………………………………………………………………………………..7

Supplementary Table S7: Sequencing primers used for colony PCR of yeast transformants……………..9

Supplementary Table S8: List of the strains……………………………………………………………………………………..10

Supplementary Figure S1…………………………………………………………………………………………………………………12

Supplementary Table S1: Details of gRNA plasmids constructed for integration into expanded Easyclone sites

| **gRNA Plasmid** | **Chromosome Number, Locus** | **20 bp guide RNA sequence** | **Source** |
| --- | --- | --- | --- |
| pCfB9336 (pgRNA_II-1_NatMX) | II, 341622…341641 | AACTGCTCAGGGCGGATAAC | This study |
| pCfB9337 (pgRNA_IV-1_NatMX) | IV, 132921…132940 | CTGCAAGGAAGTTTAAGCGT | This study |
| pCfB9339 (pgRNA_VII-1_NatMX) | VII, 438509…438490 | GTACTGCATGATATATTCAA | This study |
| pCfB9340 (pgRNA_VIII-1_NatMX) | VIII, 293615…293634 | TGTGGCCACTCGTTTAGGAA | This study |
| pCfB9341 (pgRNA_IX-1_NatMX) | IX, 388864…388883 | ATCTTAAATGAAAGACAGAG | This study |
| pCfB9342 (pgRNA_XIII-1_NatMX) | XIII, 306804…306823 | GTCACAATTCGCAGACATAT | This study |
| pCfB9343 (pgRNA_XV-1_NatMX) | XV, 93186…93205 | ATTATGAAAGTTTTCAACTA | This study |
| pCfB9344 (pgRNA_XVI-1_NatMX) | XVI, 406267…406286 | CACCATTCAAGTTACCGAGA | This study |

Supplementary Table S2: List of multiple gRNA plasmids constructed for integration into expanded Easyclone sites

| **Plasmid** | **Parent Vector** | **BioBricks** | **Source** |
| --- | --- | --- | --- |
| pCFB10157 (II-1_IV-1_gRNA_NatMX) | pTAJAK-71 (pESC-NatMXsyn-USER) | BB4840 (gRNA-1/ II_1), BB4843 (gRNA-2/ IV_1) | This study |
| pCFB10158 (VIII-1_XV-1_gRNA_NatMX) | pTAJAK-71 (pESC-NatMXsyn-USER) | BB4841 (gRNA-1/ VIII_1), BB4844 (gRNA-2/ XV_1) | This study |
| pCfB10160 (IV-1_XV-1_XVI-1_gRNA_NatMX) | pTAJAK-71 (pESC-NatMXsyn-USER) | BB4842 (gRNA-1/ IV_1), BB4847 (gRNA-2/ XV_1), BB4849 (gRNA-3/ XVI_1) | This study |
| pCfB10161 (II-1_IV-1_VIII-1_gRNA_NatMX) | pTAJAK-71 (pESC-NatMXsyn-USER) | BB4840 (gRNA-1/ II_1), BB4846 (gRNA-2/ IV_1), BB4848 (gRNA-3/ VIII_1) | This study |

Supplementary Table S3: List of backbone plasmids developed for integration into expanded Easyclone sites

| **Plasmid** | **BioBricks** | **Source** |
| --- | --- | --- |
| pCfB9355(II-1_Markerfree_BackBone) | BB4367(Vector_Backbone), BB4368 (TADH1-TCYC1), BB4371 (II-1_UP), BB4372 (II-1_DW) | This study |
| pCfB9356(IV-1_Markerfree_BackBone) | BB4367(Vector_Backbone), BB4368 (TADH1-TCYC1), BB4373 (IV-1_UP), BB4374 (IV-1_DW) | This study |
| pCfB9358(VII-1_Markerfree_BackBone) | BB4367(Vector_Backbone), BB4368 (TADH1-TCYC1), BB4377 (VII-1_UP), BB4378 (VII-1_DW) | This study |
| pCfB9359(VIII-1_Markerfree_BackBone) | BB4367(Vector_Backbone), BB4368 (TADH1-TCYC1), BB4379 (VIII-1_UP), BB4380 (VIII-1_DW) | This study |
| pCfB9360(IX-1_Markerfree_BackBone) | BB4367(Vector_Backbone), BB4368 (TADH1-TCYC1), BB4381 (IX-1_UP), BB4382 (IX-1_DW) | This study |
| pCfB9361(XIII-1_Markerfree_BackBone) | BB4367(Vector_Backbone), BB4368 (TADH1-TCYC1), BB4383 (XIII-1_UP), BB4384 (XIII-1_Dw) | This study |
| pCfB9362(XV-1_Markerfree_BackBone) | BB4367(Vector_Backbone), BB4368 (TADH1-TCYC1), BB4385 (XV-1_UP), BB4386 (XV-1_Dw) | This study |
| pCfB9363(XVI-1_Markerfree_BackBone) | BB4367(Vector_Backbone), BB4368 (TADH1-TCYC1), BB4387 (XVI-1_UP), BB4388 (XVI-1_Dw) | This study |

Supplementary Table S4: List of integrative plasmids for gfp integration into expanded Easyclone sites

| **Plasmid** | **Parent Vector** | **BioBricks** | **Source** |
| --- | --- | --- | --- |
| pCfB9365 (II-1_Ptef1-gfp-Tadh1) | pCfB9355(II-1_Markerfree_BackBone) | [BB0008 (PTEF1<-)](javascript:void(0)), [BB4685 (GFP_U1)](javascript:void(0)) | This study |
| pCfB9366 (IV-1_Ptef1-gfp-Tadh1) | pCfB9356(IV-1_Markerfree_BackBone) | [BB0008 (PTEF1<-)](javascript:void(0)), [BB4685 (GFP_U1)](javascript:void(0)) | This study |
| pCfB9368 (VII-1_Ptef1-gfp-Tadh1) | pCfB9358(VII-1_Markerfree_BackBone) | [BB0008 (PTEF1<-)](javascript:void(0)), [BB4685 (GFP_U1)](javascript:void(0)) | This study |
| pCfB9369 (VIII-1_Ptef1-gfp-Tadh1) | pCfB9359(VIII-1_Markerfree_BackBone) | [BB0008 (PTEF1<-)](javascript:void(0)), [BB4685 (GFP_U1)](javascript:void(0)) | This study |
| pCfB9370 (IX-1_Ptef1-gfp-Tadh1) | pCfB9360(IX-1_Markerfree_BackBone) | [BB0008 (PTEF1<-)](javascript:void(0)), [BB4685 (GFP_U1)](javascript:void(0)) | This study |
| pCfB9371 (XIII-1_Ptef1-gfp-Tadh1) | pCfB9361(XIII-1_Markerfree_BackBone) | [BB0008 (PTEF1<-)](javascript:void(0)), [BB4685 (GFP_U1)](javascript:void(0)) | This study |
| pCfB9372 (XV-1_Ptef1-gfp-Tadh1) | pCfB9362(XV-1_Markerfree_BackBone) | [BB0008 (PTEF1<-)](javascript:void(0)), [BB4685 (GFP_U1)](javascript:void(0)) | This study |
| pCfB9373 (XVI-1_Ptef1-gfp-Tadh1) | pCfB9363(XVI-1_Markerfree_BackBone) | [BB0008 (PTEF1<-)](javascript:void(0)), [BB4685 (GFP_U1)](javascript:void(0)) | This study |
| pCfB10514 (II-1_Ppgk1-gfp-Tcyc1) | pCfB9355(II-1_Markerfree_BackBone) | BB0009 (PPGK1->), BB5107 (GFP_U2) | This study |
| pCfB10515 (IV-1_Ppgk1-gfp-Tcyc1) | pCfB9356(IV-1_Markerfree_BackBone) | BB0009 (PPGK1->), BB5107 (GFP_U2) | This study |
| pCfB10516 (VII-1_Ppgk1-gfp-Tcyc1) | pCfB9358(VII-1_Markerfree_BackBone) | BB0009 (PPGK1->), BB5107 (GFP_U2) | This study |
| pCfB10517 (VIII-1_Ppgk1-gfp-Tcyc1) | pCfB9359(VIII-1_Markerfree_BackBone) | BB0009 (PPGK1->), BB5107 (GFP_U2) | This study |
| pCfB10518 (IX-1_Ppgk1-gfp-Tcyc1) | pCfB9360(IX-1_Markerfree_BackBone) | BB0009 (PPGK1->), BB5107 (GFP_U2) | This study |
| pCfB10519 (XIII-1_Ppgk1-gfp-Tcyc1) | pCfB9361(XIII-1_Markerfree_BackBone) | BB0009 (PPGK1->), BB5107 (GFP_U2) | This study |
| pCfB10520 (XV-1_Ppgk1-gfp-Tcyc1) | pCfB9362(XV-1_Markerfree_BackBone) | BB0009 (PPGK1->), BB5107 (GFP_U2) | This study |
| pCfB10521 (XVI-1_Ppgk1-gfp-Tcyc1) | pCfB9363(XVI-1_Markerfree_BackBone) | BB0009 (PPGK1->), BB5107 (GFP_U2) | This study |

Supplementary Table S5: List of BioBricks used in this study

| **BioBrick** | **PCR template** | **Forward primer** | **Reverse primer** | **Source** |
| --- | --- | --- | --- | --- |
| [BB0008 (PTEF1<-)](javascript:void(0)) | pCfB0029 | 5 | 6 | ^1^ |
| BB0009 (PPGK1->) | pCfB0029 | 7 | 8 | ^1^ |
| BB4367(Vector_Backbone) | pCfB2899 (X-2-MarkerFree) | 11110 | 11111 | This study |
| BB4368 (TADH1-TCYC1) | pCfB2899 (X-2-MarkerFree) | 26341 | 26342 | This study |
| BB4371 (II-1_UP) | gDNA of CEN.PK | 26347 | 26348 | This study |
| BB4372 (II-1_DW) | gDNA of CEN.PK | 26349 | 26350 | This study |
| BB4373 (IV-1_UP) | gDNA of CEN.PK | 26351 | 26352 | This study |
| BB4374 (IV-1_DW) | gDNA of CEN.PK | 26353 | 26354 | This study |
| BB4377 (VII-1_UP) | gDNA of CEN.PK | 26359 | 26360 | This study |
| BB4378 (VII-1_DW) | gDNA of CEN.PK | 26361 | 26362 | This study |
| BB4379 (VIII-1_UP) | gDNA of CEN.PK | 26363 | 26364 | This study |
| BB4380 (VIII-1_DW) | gDNA of CEN.PK | 26365 | 26366 | This study |
| BB4381 (IX-1_UP) | gDNA of CEN.PK | 26367 | 26368 | This study |
| BB4382 (IX-1_DW) | gDNA of CEN.PK | 26369 | 26370 | This study |
| BB4383 (XIII-1_UP) | gDNA of CEN.PK | 26371 | 26372 | This study |
| BB4384 (XIII-1_Dw) | gDNA of CEN.PK | 26373 | 26374 | This study |
| BB4385 (XV-1_UP) | gDNA of CEN.PK | 26375 | 26376 | This study |
| BB4386 (XV-1_Dw) | gDNA of CEN.PK | 26377 | 26378 | This study |
| BB4387 (XVI-1_UP) | gDNA of CEN.PK | 26379 | 26380 | This study |
| BB4388 (XVI-1_Dw) | gDNA of CEN.PK | 26381 | 26382 | This study |
| [BB4685 (GFP_U1)](javascript:void(0)) | pCfB9024 | 27392 | 27393 | This study |
| BB4840 (gRNA-1/ II_1) | pCfB9336 (pgRNA_II-1_NatMX) | 10525 | 10530 | This study |
| BB4841 (gRNA-1/ VIII_1) | pCfB9340 (pgRNA_VIII-1_NatMX) | 10525 | 10530 | This study |
| BB4842 (gRNA-1/ IV_1) | pCfB9340 (pgRNA_VIII-1_NatMX) | 10525 | 10530 | This study |
| BB4843 (gRNA-2/ IV_1) | pCfB9340 (pgRNA_VIII-1_NatMX) | 10526 | 10529 | This study |
| BB4844 (gRNA-2/ XV_1) | pCfB9343 (pgRNA_XV-1_NatMX) | 10526 | 10529 | This study |
| BB4846 (gRNA-2/ IV_1) | pCfB9337 (pgRNA_IV-1_NatMX) | 10526 | 10531 | This study |
| BB4847 (gRNA-2/ XV_1) | pCfB9343 (pgRNA_XV-1_NatMX) | 10526 | 10531 | This study |
| BB4848 (gRNA-3/ VIII_1) | pCfB9340 (pgRNA_VIII-1_NatMX) | 10527 | 10529 | This study |
| BB4849 (gRNA-3/ XVI_1) | pCfB9340 (pgRNA_VIII-1_NatMX) | 10527 | 10529 | This study |
| BB5107 (GFP_U2) | pCfB9024 | 28373 | 24465 | This study |

Supplementary Table S6: List of Primers used in PCR amplifications in this study. The USER overhang sequence is in lowercase.

| **Primer** | **Sequence (5’-> 3’)** |
| --- | --- |
| PR-5 | acctgcacu TTGTAATTAAAACTTAG |
| PR-6 | cacgcgau GCACACACCATAGCTTC |
| PR-7 | cgtgcgau GGAAGTACCTTCAAAGA |
| PR-8 | atgacagau TTGTTTTATATTTGTTG |
| PR-2221 | GTTGACACTTCTAAATAAGCGAATTTC |
| PR-10525 | cgtgcgauAGGGAACAAAAGCTGGAGCT |
| PR-10526 | agtgcagguAGGGAACAAAAGCTGGAGCT |
| PR-10527 | atctgtcauAGGGAACAAAAGCTGGAGCT |
| PR-10529 | cacgcgauTAACTAATTACATGACTCGA |
| PR-10530 | acctgcacuTAACTAATTACATGACTCGA |
| PR-10531 | atgacagauTAACTAATTACATGACTCGA |
| PR-11110 (E.coli backboneUSER _fw) | ATCGCGTGCATTCGCGGCCGCATTTAAATCC |
| PR-11111 (E.coli backboneUSER _rev) | ATCGCACGCATTCGCGGCCGCAAATTTAAATAAAATG |
| PR-24465 (GFP_U2_rev) | cacgcgaUTTATTTGTACAATTCATCCATACC |
| PR-26341 (TADH1-TCYC1_fw) | acgtgcaacgctU GAG CGA CCT CAT GCT ATA |
| PR-26342 (TADH1-TCYC1_rev) | aggccacU CTT CGA GCG TCC CAA AA |
| PR-26347(II-1_UP_fw) | cgtgcgaU AGGTATTAAAAAGGGACATACT |
| PR-26348(II-1_UP_rev) | aagcgttgcacgU AAAATAACATGTTGCGTGC |
| PR-26349(II-1_Dw_fw) | agtggccU CTG AGC AGT TTT CTT CCC |
| PR-26350(II-1_Dw_rev) | cacgcgaU TAT GCC GTG ATA TGA ACA A |
| PR-26351(IV-1_UP_fw) | cgtgcgaU ATC TCT CCA AGG TTA GCC |
| PR-26352(IV-1_UP_rev) | aagcgttgcacgU AGA GTT CCC GTC GGA AT |
| PR-26353(IV-1_Dw_fw) | agtggccU TTA CTT TGC TAG GTT GAG |
| PR-26354(IV-1_Dw_rev) | cacgcgaU GAT TTG GTT TAG CAG CAG |
| PR-26359(VII-1_UP_fw) | cgtgcgaU TCT GCC TGG GTA GAT TTT |
| PR-26360(VII-1_UP_rev) | aagcgttgcacgU ATG GTG TGT ATT TTA GTA CG |
| PR-26361(VII-1_Dw_fw) | agtggccU CAT GCA GTA CAT TGA CAG TA |
| PR-26362(VII-1_Dw_rev) | cacgcgaU TCC GGT CTG TTT GGT TAT A |
| PR-26363(VIII-1_UP_fw) | cgtgcgaU TTG ACT CAA TGC AAC AGT |
| PR-26364(VIII-1_UP_rev) | aagcgttgcacgU GGC CAC AAT GAA ACT TC |
| PR-26365(VIII-1_Dw_fw) | agtggccU AAT AAC CTC CGC GAA TG |
| PR-26366(VIII-1_Dw_rev) | cacgcgaU AAA CTC CGC TAC CTG |
| PR-26367(X-1_UP_fw) | cgtgcgaU CTCTGTGATCTTCTAAGATA |
| PR-26368(IX-1_UP_rev) | aagcgttgcacgU TCG CGA GAT AGA ACG AC |
| PR-26369(IX-1_Dw_fw) | agtggccU TTG ATG ACA CTA GCG GA |
| PR-26370(X-1_Dw_rev) | cacgcgaU AAC TAC CCG TAG AAT ACA T |
| PR-26371(XIII-1_UP_fw) | cgtgcgaU TCC TTC TAC TGC GGT TT |
| PR-26372(XIII-1_UP_rev) | aagcgttgcacgU AAT TGT GAC AAC CAA TCC |
| PR-26373(XIII-1_Dw_fw) | agtggccU CAA TGT TTC CTC CCC T |
| PR-26374(XIII-1_Dw_rev) | cacgcgaU TAC TGT GGT GAA TCT TAT GC |
| PR-26375(XV-1_UP_fw) | cgtgcgaU ATT GAT TTA ACC ATG GGT |
| PR-26376(XV-1_UP_rev) | aagcgttgcacgU CTT CCG TTT TCA ATC TTC |
| PR-26377(XV-1_Dw_fw) | agtggccU TCT AGC CTA TTG ATG GTG |
| PR-26378(XV-1_Dw_rev) | cacgcgaU GTC GTT GTT GGT ACT GTT A |
| PR-26379(XVI-1_UP_fw) | cgtgcgaU TTA GCC TTG AAG GTC G |
| PR-26380(XVI-1_UP_rev) | aagcgttgcacgU GGT GAC AAA TAT GTC GC |
| PR-26381(XVI-1_Dw_fw) | agtggccU TAG AGA GGT GGA TGG CT |
| PR-26382(XVI-1_Dw_rev) | cacgcgaU CAG TAG CAA AGT ATT GTC G |
| [PR-27392 (GFP_U1_U1_fw)](javascript:void(0)) | agtgcaggU AAAACA ATG TCT AAA GGT GAA GAA TTA TTC AC |
| PR-27393 (GFP_U1_U1_rev) | cgtgcgaU TTA TTT GTA CAA TTC ATC CAT ACC |
| PR-26989(II-1_ycPCR_fw) | TCCTTATCCATTCAGCTTCTC |
| PR-26990(II-1_ycPCR_rev) | ACC CTT TTT TTC CAT TCC CTC |
| PR-26991(IV-1_ycPCR_fw) | CCG TCA AGT CCA TCC ATC C |
| PR-26992(IV-1_ycPCR_rev) | GTT CAC CTT CTC TCA GCG |
| PR-26995(VII-1_ycPCR_fw) | CCA AAG AAG CAC CGG TAA |
| PR-26996(VII-1_ycPCR_rev) | CTG GGG CCT GTT GAA ATG |
| PR-26997(VIII-1_ycPCR_fw) | TGCGCATATTTGTCAGTCCT |
| PR-26998(VIII-1_ycPCR_rev) | ACT TTT CTG CCC ACT TCT CT |
| PR-26999(IX-1_ycPCR_fw) | ACAGTGAGAGAGTGGCAT |
| PR-27000(IX-1_ycPCR_rev) | ACT TAC CAA GGT GCT GCT |
| PR-27001(XIII-1_ycPCR_fw) | GCCCACGGAAGAAAAAGC |
| PR-27002(XIII-1_ycPCR_rev) | CAC GTC TTC TGC TTC CTG |
| PR-27003(XV-1_ycPCR_fw) | AGC ACC ACT ACC CTT AAT CT |
| PR-27004(XV-1_ycPCR_rev) | ACT ATT GTT TTC CGG TCC TTC G |
| PR-27005(XVI-1_ycPCR_fw) | ACA AGA GAC GAA AAG GAC CC |
| PR-27006(XVI-1_ycPCR_rev) | CAC TTG CTT TGC TCA CTA CTC |
| PR-28373 (GFP_U2_fw) | atctgtcaU AAAACA ATGTCT AAA GGT GAA GAA TTA TTC AC |

Supplementary Table S7: Sequencing primers used for colony PCR of yeast transformants.

The primer 1 and 2 bind to regions in homologous arms of UP and DW of integration site, while PR-2221 (primer 3) binds to T*adh1*. Depending on the primers bound to genomic DNA of yeast transformants used as template, the correct or incorrect size of fragment is obtained. An example of the PCR results for integration sites is shown below.

| **Integration Site** | **Primers** | | | **Size (bp)** | |
| --- | --- | --- | --- | --- | --- |
|  | **1** | **2** | **3** | **Correct** | **Incorrect** |
| II-1 | PR-26989 | PR-26990 | PR-2221 | 800 | 1251 |
| IV-1 | PR-26991 | PR-26992 | PR-2221 | 1085 | 1508 |
| VII-1 | PR-26995 | PR-26996 | PR-2221 | 1006 | 1658 |
| VIII-1 | PR-26997 | PR-26998 | PR-2221 | 774 | 1292 |
| IX-1 | PR-26999 | PR-27000 | PR-2221 | 890 | 1476 |
| XIII-1 | PR-27001 | PR-27002 | PR-2221 | 858 | 1517 |
| XV-1 | PR-27003 | PR-27004 | PR-2221 | 800 | 1328 |
| XVI-1 | PR-27005 | PR-27006 | PR-2221 | 840 | 1276 |


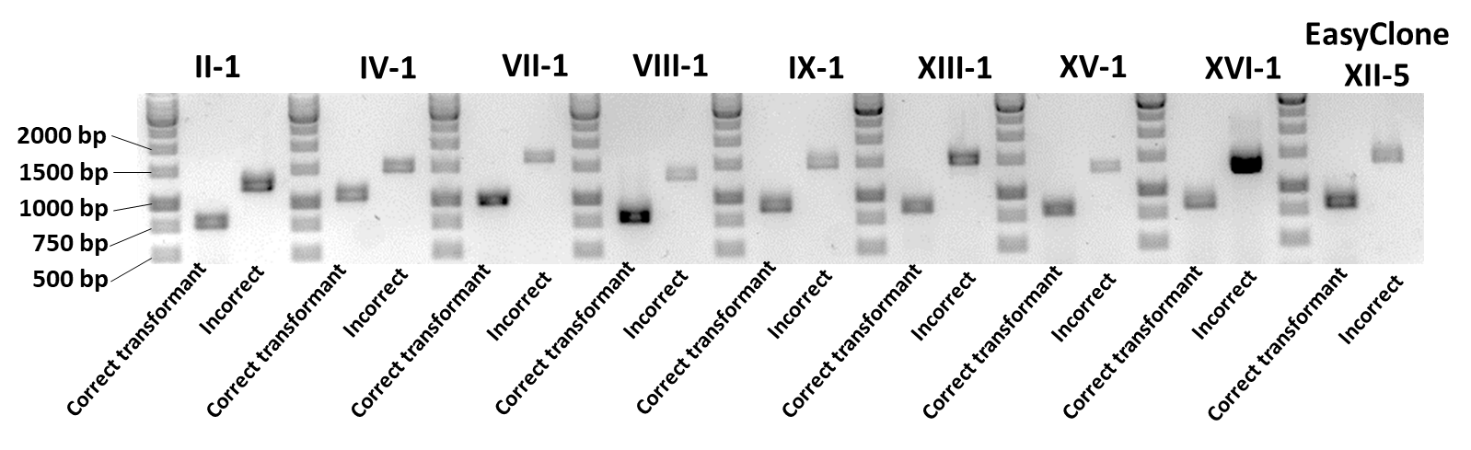


Supplementary Table S8: List of the strains constructed in this study

| **Name** | **Parent Strain** | **Added DNA Element(s)** | **gRNA vector** | **Relevant Genotype** | **Source** |
| --- | --- | --- | --- | --- | --- |
| CEN.PK 113-7D |  |  |  | MATa *MAL2-8c SUC2 URA3 HIS3 LEU2 TRP1* | Peter Kötter |
| ST7574 | CEN.PK 113-7D | pCfB2312 |  | CEN.PK113-7D ↑Cas9_KanMX | ^2^ |
| ST9978 | ST7574 | pCfB9365 (II-1_Ptef1-gfp-Tadh1) | pCfB9336 | CEN.PK113-7D ↑Cas9_KanMX chrII_↑P*tef1*-*GFP-*T*adh1* | This study |
| ST9979 | ST7574 | pCfB9366 (IV-1_Ptef1-gfp-Tadh1) | pCfB9337 | CEN.PK113-7D ↑Cas9_KanMX chrIV_↑P*tef1*-*GFP-*T*adh1* | This study |
| ST9981 | ST7574 | pCfB9368 (VII-1_Ptef1-gfp-Tadh1) | pCfB9339 | CEN.PK113-7D ↑Cas9_KanMX chrVII_↑P*tef1*-*GFP-*T*adh1* | This study |
| ST9982 | ST7574 | pCfB9369 (VIII-1_Ptef1-gfp-Tadh1) | pCfB9340 | CEN.PK113-7D ↑Cas9_KanMX chrVIII_↑P*tef1*-*GFP-*T*adh1* | This study |
| ST9983 | ST7574 | pCfB9370 (IX-1_Ptef1-gfp-Tadh1) | pCfB9341 | CEN.PK113-7D ↑Cas9_KanMX chrIX_↑P*tef1*-*GFP-*T*adh1* | This study |
| ST9984 | ST7574 | pCfB9371 (XIII-1_Ptef1-gfp-Tadh1) | pCfB9342 | CEN.PK113-7D ↑Cas9_KanMX chrXIII_↑P*tef1*-*GFP-*T*adh1* | This study |
| ST9985 | ST7574 | pCfB9372 (XV-1_Ptef1-gfp-Tadh1) | pCfB9343 | CEN.PK113-7D ↑Cas9_KanMX chrXV_↑P*tef1*-*GFP-*T*adh1* | This study |
| ST9986 | ST7574 | pCfB9373 (XVI-1_Ptef1-gfp-Tadh1) | pCfB9344 | CEN.PK113-7D ↑Cas9_KanMX chrXVI_↑P*tef1*-*GFP-*T*adh1* | This study |
| ST10937 | ST7574 | pCfB9365 (II-1_Ptef1-gfp-Tadh1), pCfB9366 (IV-1_Ptef1-gfp-Tadh1) | pCfB10157 | CEN.PK113-7D ↑Cas9_KanMX chrII_↑P*tef1*-*GFP-*T*adh1*, chrIV_↑P*tef1*-*GFP-*T*adh1* | This study |
| ST10938 | ST7574 | pCfB9369 (VIII-1_Ptef1-gfp-Tadh1), pCfB9372 (XV-1_Ptef1-gfp-Tadh1) | pCfB10158 | CEN.PK113-7D ↑Cas9_KanMX chrVIII_↑P*tef1*-*GFP-*T*adh1*, chrXV_↑P*tef1*-*GFP-*T*adh1* | This study |
| ST10939 | ST7574 | pCfB9366 (IV-1_Ptef1-gfp-Tadh1), pCfB9372 (XV-1_Ptef1-gfp-Tadh1), pCfB9373 (XVI-1_Ptef1-gfp-Tadh1) | pCfB10160 | CEN.PK113-7D ↑Cas9_KanMX chrIV_↑P*tef1*-*GFP-*T*adh1*, chrXV_↑P*tef1*-*GFP-*T*adh1*, chrXVI_↑P*tef1*-*GFP-*T*adh1* | This study |
| ST10940 | ST7574 | pCfB9365 (II-1_Ptef1-gfp-Tadh1), pCfB9366 (IV-1_Ptef1-gfp-Tadh1), pCfB9369 (VIII-1_Ptef1-gfp-Tadh1) | pCfB10161 | CEN.PK113-7D ↑Cas9_KanMX chrII_↑P*tef1*-*GFP-*T*adh1*, chrIV_↑P*tef1*-*GFP-*T*adh1*, chrVIII_↑P*tef1*-*GFP-*T*adh1* | This study |
| ST11077 | ST7574 | pCfB10514 (II-1_Ppgk1-gfp-Tcyc1) | pCfB9336 | CEN.PK113-7D ↑Cas9_KanMX chrII_↑P*pgk1*-*GFP-*T*cyc1* | This study |
| ST11078 | ST7574 | pCfB10515 (IV-1_Ppgk1-gfp-Tcyc1) | pCfB9337 | CEN.PK113-7D ↑Cas9_KanMX chrIV_↑P*pgk1*-*GFP-*T*cyc1* | This study |
| ST11079 | ST7574 | pCfB10516 (VII-1_Ppgk1-gfp-Tcyc1) | pCfB9339 | CEN.PK113-7D ↑Cas9_KanMX chrVII_↑P*pgk1*-*GFP-*T*cyc1* | This study |
| ST11080 | ST7574 | pCfB10517 (VIII-1_Ppgk1-gfp-Tcyc1) | pCfB9340 | CEN.PK113-7D ↑Cas9_KanMX chrVIII_↑P*pgk1*-*GFP-*T*cyc1* | This study |
| ST11081 | ST7574 | pCfB10518 (IX-1_Ppgk1-gfp-Tcyc1) | pCfB9341 | CEN.PK113-7D ↑Cas9_KanMX chrIX_↑P*pgk1*-*GFP-*T*cyc1* | This study |
| ST11082 | ST7574 | pCfB10519 (XIII-1_Ppgk1-gfp-Tcyc1) | pCfB9342 | CEN.PK113-7D ↑Cas9_KanMX chrXIII_↑P*pgk1*-*GFP-*T*cyc1* | This study |
| ST11083 | ST7574 | pCfB10520 (XV-1_Ppgk1-gfp-Tcyc1) | pCfB9343 | CEN.PK113-7D ↑Cas9_KanMX chrXV_↑P*pgk1*-*GFP-*T*cyc1* | This study |
| ST11084 | ST7574 | pCfB10521 (XVI-1_Ppgk1-gfp-Tcyc1) | pCfB9344 | CEN.PK113-7D ↑Cas9_KanMX chrXVI_↑P*pgk1*-*GFP-*T*cyc1* | This study |


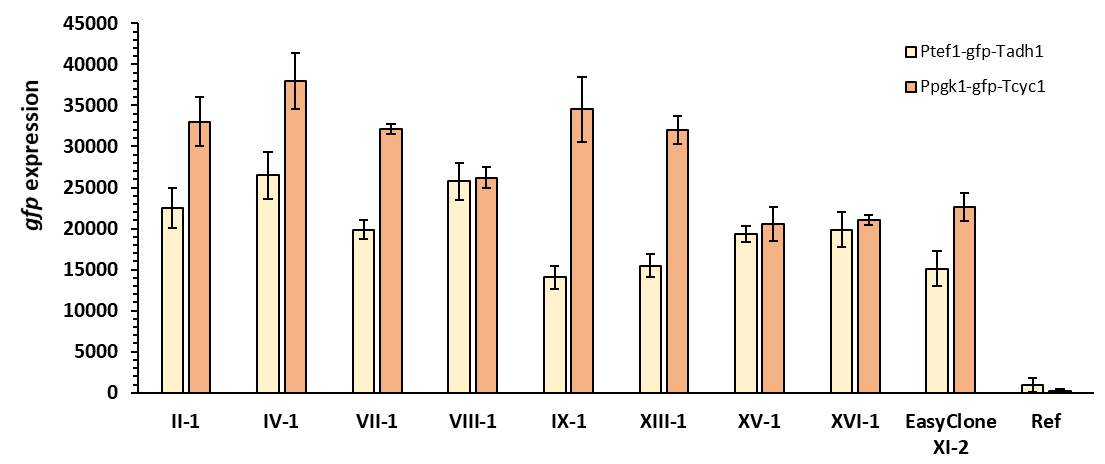


Supplementary Figure S1: Comparison of *gfp* expression level for the two constructs P*tef1-gfp-*T*adh1* and P*pgk1-gfp*-T*cyc1* integrated into expanded EasyClone sites, the values are for cells after cultivation for ca. 60 generations. Expression from EasyClone site XI-2 is shown for comparison.

**Supplementary References:**

(1) Rodriguez, A., Kildegaard, K. R., Li, M., Borodina, I., and Nielsen, J. (2015) Establishment of a yeast platform strain for production of p-coumaric acid through metabolic engineering of aromatic amino acid biosynthesis. *Metab. Eng.* *31*, 181–188.

(2) van der Hoek, S. A., Darbani, B., Zugaj, K. E., Prabhala, B. K., Biron, M. B., Randelovic, M., Medina, J. B., Kell, D. B., and Borodina, I. (2019) Engineering the Yeast Saccharomyces cerevisiae for the Production of L-(+)-Ergothioneine. *Front Bioeng Biotechnol* *7*, 262.
